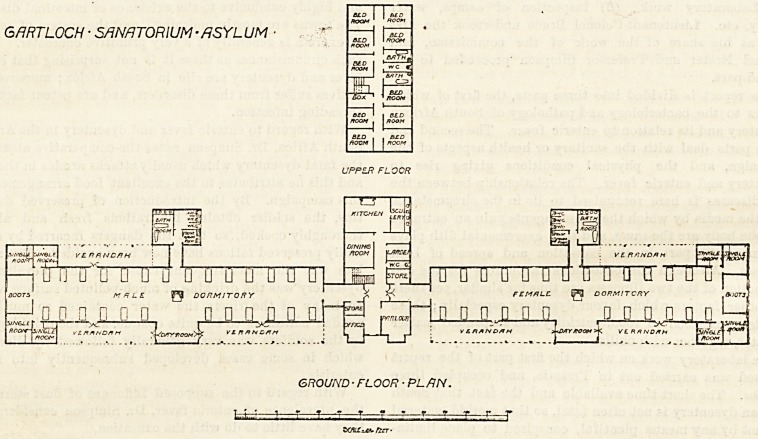# Sanatorium for Consumptive Patients at the Gartloch Asylum

**Published:** 1903-08-22

**Authors:** 


### HOSPITAL ADMINISTRATION.

### CONSTRUCTION AND ECONOMICS.

### SANATORIUM FOR CONSUMPTIVE PATIENTS AT THE GARTLOCH ASYLUM.

It has been known for a long time that phthisis was more common in lunatic asylums than in the population generally. For some years the question has been a pressing one and has led to much discussion among medical superintendents who have advocated the separation of these consumptive lunatics from the rest of their patients. In some asylums this separation has been carried out as thoroughly as existing conditions permitted the segregation without adding new buildings; but manifestly the only perfectly satisfactory course is to place these phthisical patients in specially constructed annexes.

This has been done by the Glasgow District Lunacy Board at the asylums of Woodilee and Gartloch, and we publish herewith the plans of the sanatorium erected at the latter asylum. It consists of an administrative centre and two wings. The centre contains sitting-rooms, dining-room and kitchen, etc., on the ground floor, and on the first floor 10 bedrooms, all for the nursing staff. Short corridors connect the administrative centre with the wards. Each ward contains 26 beds, and there is on the south side a large square bay which is used as a day-room, and from which access is obtained to the verandahs extending along the whole of the south front and part of the north front. Opposite the bay is the sanitary annexe which is very well planned and is effectually cut off from the main by a cross-ventilated corridor. At the free end of the ward are four single-bedded rooms, two on each side, and a boot-porch. It is often a difficult problem to construct these single-bedded rooms so that they shall be at once easy of access, that noise in them shall not be heard in the ward, and that they shall have efficient cross-ventilation. So far as can be judged from the plans we doubt if the latter has been obtained here, but it is probable that fanlights are placed over the doors of these rooms, and if so these will do something to secure that essential cross-current. Single-bedded rooms are valuable in asylums, but even remembering this fact it would have been better to omit two of these rooms, in which case cross-ventilation of the remaining two would have been easy, and the two end beds in the ward would have been much better placed. As it is we should not like to use these end beds. They are too far from the windows. Apart from these little blemishes it may be truly said that the design is a good one, and that it may be accepted as a model. The accommodation is for 60 patients. The wards are of one story only, and the administrative centre is of two stories. The front elevation faces due south, extends to about 400 feet, and is carried out in a very pleasing manner somewhat resembling the old English half-timbered style of architecture.

As an essential point in a consumption hospital it should be mentioned that the upper window sashes open by falling inwards, and the lower part opens outward in two glazed doors, which can only be opened or closed by a carriage key, and this very proper arrangement puts it out of a patient's power to interfere with the influx of air.

The sanatorium is constructed of iron and wood, with an air space between, and the inner surface of the walls is lined with a jointless and non-porous composition which is said to be "a perfect sanitary medium." All angles and corners are rounded off.

The building proper, which, by the way, is said to be practically fireproof, would seem to have cost about £90 a bed; but if navvy work, electric lighting, water and sewage works, road-making, and furnishing be included, the cost rises to £143 a bed.

The building was put up by Messrs. Spiers and Co., of Glasgow, on their patent system.

## Figures and Tables

**Figure f1:**